# Supplementary material for: A mouse-tracking study of the composite nature of the Stroop effect at the level of response execution
Source: PLoS One. 2023 Jan 19;18(1):e0279036. doi: 10.1371/journal.pone.0279036 (PMC9851562; doi:10.1371/journal.pone.0279036)
Supplement: S1 File — Description of the data analysis method with linear mixed models, including the model selection procedure. (PDF) [file pone.0279036.s001.pdf]

S1. Linear Mixed Modelling method

As indicated in the main text, Linear Mixed Modelling (LMM) approach with the lme4 R package (v1.1.23, [1]) was used for fitting regression models of the data. From the fitted regression equations, the marginal means of each experimental conditions were estimated and differences across conditions corresponding to the overall Stroop effect, Stroop interference and facilitation and their respective decompositions were tested. For modelling purposes, the six experimental conditions were recoded into dummy variables with the colour-neutral word condition (e.g., $\mathit{CARGO}_{\text{blue}}$) as reference level. In all of the models, five resulting fixed parameters were included. In order to select a parsimonious random effect structure, a general method derived from Bates et al. [2] and Matuschek et al. [3] was followed.

In a first step, the maximal random effect structure was tested for overfitting using a Principal Component Analysis (PCA) method (see [2]). If there was evidence of overfitting, in a second step, all the random correlations were dropped, and the lowest variance parameters (random slopes) were trimmed until the model was correctly specified (based on the PCA). In this step, the relevance of the remaining low variance parameters was tested using likelihood ratio tests (LRT) – starting with the lowest variance. These parameters were dropped if they did not improve the model's fit significantly. In a third step, the correlation between the remaining random slopes were re-included. Overfitting was tested again using the PCA and low variance parameters were removed until the model was well specified. In a fourth and final step, the LRT was used to trim low variance random slopes and low random correlations. This procedure was applied manually for the initiation times, response times and maximal deviation models. Since one model was fitted per normalized time coordinate, this procedure had to be automated for the analysis of deviation as a function of time. Therefore, to obtain more consistent model structure from one time step to the next, the fourth step was simplified. At this step, the algorithm trimmed the low variance parameters but not the random correlations involving each slope (and intercept) individually. Instead, after trimming the low variance parameters, the model including all the correlations between the remaining random parameters was tested against the model

without correlations. To this end, LRT was used to estimate whether the former improved the fit significantly compared with the latter.

Following Matushek et al. [3], for LRT the alpha value was set to 0.2. It allows for selecting slightly more complex models (as compared to a model selection based on the Akaike Information Criterion (AIC) or a more common alpha value of 0.05) and thus for slightly more conservative analyses while reducing the statistical power slightly [3,4]. However, compared with the model including the maximal random effect structure, the final models are correctly parametrized given the available data (each parameter can be fitted accurately). Recall that the alpha value should be understood as a model selection criterion allowing a selection of more or less complex models more than as a null hypothesis significance testing. For instance, Matushek et al. [3] indicate that in their own dataset, using the AIC for model selection was equivalent to using the LRT with an alpha value of around 0.15.

References

[1] Bates D, Mächler M, Bolker B, Walker S. Fitting linear mixed-effects models using lme4. Journal of Statistical Software 2015;067.
[2] Bates D, Kliegl R, Vasishth S, Baayen H. Parsimonious mixed models. arXiv; 2018. https://doi.org/10.48550/arXiv.1506.04967.
[3] Matuschek H, Kliegl R, Vasishth S, Baayen H, Bates D. Balancing Type I error and power in linear mixed models. Journal of Memory and Language 2017;94:305–15. https://doi.org/10.1016/j.jml.2017.01.001.
[4] Barr DJ, Levy R, Scheepers C, Tily HJ. Random effects structure for confirmatory hypothesis testing: Keep it maximal. Journal of Memory and Language 2013;68:255–78. https://doi.org/10.1016/j.jml.2012.11.001.
