# Supplementary material for: A mouse-tracking study of the composite nature of the Stroop effect at the level of response execution
Source: PLoS One. 2023 Jan 19;18(1):e0279036. doi: 10.1371/journal.pone.0279036 (PMC9851562; doi:10.1371/journal.pone.0279036)
Supplement: S2 File — Complete report of the fitted linear mixed models’ parameters. (PDF) [file pone.0279036.s002.pdf]

S2. Linear mixed models' fixed and random fitted parameters

The main text contains no details about the parameters fitted for estimating the statistical models. For clarity reasons, the differences between marginal means estimated from the fitted model were presented instead. The present section reports the parameters fitted for the initiation times, response times and maximal deviation models. Fixed parameters represent the intercept and slopes of each dummy variable in the regression equation (dummy variables contrasting colour-neutral stimuli from the other stimulus categories). Random parameters represent the variance around the intercept and the slopes across participants. Random correlation represents how intercept and slopes covary at the level of the participants random variable. In the random effects section, letters in parentheses index the parameters' column in the random correlation matrices when relevant.

Initiation times

Fitted model.

After parsimonious random structure selection procedure, random intercept was the only random parameter selected. The unit of the fixed parameters is in milliseconds.

Random effects:

**Groups**	**Name**	**Variance**	**Std.Dev.**
Participants	(Intercept)	4271	65.35
Residual		12302	110.92

Number of observations: 15619. Grouping variable: participants, 83

Fixed effects:

	**Estimate**	**Std. Error**	**t-value**
(Intercept)	168.49019	7.49279	22.487
Non-response set	0.02672	3.06755	0.009
Associated color-inc	-0.89199	3.06546	-0.291
Standard color-inc	0.88632	3.08461	0.287
Associated color-cong	1.09000	3.06546	0.356
Standard color-cong	-1.63305	3.06427	-0.533

Stroop decomposition (Pairwise differences between conditions' marginal means)

This section presents the components of the Stroop decomposition as estimated from the above model. These statistics are presented in the Results section of the main text for the response times, maximal mouse deviation and partial errors (see also Figures 4, 5 and 6).

Contrast	estimate (SE)	df	Lower CI	Higher CI
**The total Stroop effect:**				
**Standard color-inc – std. Color-cong**	**2.519 (3.09)**	**15531**	**-3.53**	**8.57**
**Overall Interference:**				
**Standard color-inc – color-neutral**	**0.886 (3.08)**	**15531**	**-5.16**	**6.93**
Response conflict:				
Standard color-inc – non-response set	0.86 (3.09)	15531	-5.20	6.92
Semantic conflict:				
Associated color-inc – color-neutral	-0.892 (3.07)	15531	-6.90	5.12
Semantic relevance:				
Color-neutral – associated color-inc	0.919 (3.07)	15531	-5.10	6.94
**Overall facilitation:**				
**Color-neutral – standard color-cong**	**1.633 (3.06)**	**15531**	**-4.37**	**7.64**
Response facilitation:				
Associated color-cong – std. Color-cong	2.723 (3.07)	15531	-3.29	8.74
Semantic facilitation:				
Color-neutral – associated color-cong	1.09 (3.07)	15531	-4.92	7.10

Response times

After selecting a parsimonious random structure, the random intercept was kept as well as slopes estimating the difference between colour-neutral stimuli on one hand, and non-response set and standard colour-incongruent stimuli on the other hand. All random correlation were estimated. The unit of the fixed parameters is in milliseconds.

Random effects:

Groups	Name	Variance	Std.Dev.	Random corr. matrix	
Participants	(Intercept) (a)	12785.3	113.07	(a)	(b)
	Non-response set (b)	908.8	30.15	0.45	
	Standard color-inc	9545.8	97.70	0.25	0.56
Residual		51927.7	227.88		

Number of observations: 15619. Grouping variable: participants, 83

Fixed effects:

	**Estimate**	**Std. Error**	**t value**
(Intercept)	1070.102	13.184	81.166
Non-response set	41.319	7.118	5.805
Associated color-inc	4.508	6.298	0.716
Standard color-inc	119.192	12.458	9.568
Associated color-cong	-24.999	6.298	-3.969
Standard color-cong	-40.643	6.296	-6.456

Maximal deviation:

After selecting a parsimonious random structure, the random intercept was kept as well as slopes estimating the difference between colour-neutral stimuli on one hand, and non-response set, standard colour-incongruent and associated colour-incongruent stimuli on the other hand. Random correlations between the intercept and the random slopes were discarded. Random correlations between random slopes were included in the model. The unit of the fixed parameters depends on the spatial coordinates scale after rightward remapping and space-normalizing the mouse trajectories (x: [0,1]; y: [0,1.5]).

Random effects:

**Groups**	**Name**	**Variance**	**Std.Dev.**	**Random corr. matrix**	
participants	(Intercept)	0.019691	0.14033		
participants.1	Non-response set (a)	0.004237	0.06509	(a)	(b)
	Associated color-inc (b)	0.006538	0.08086	0.71	
	Standard color-inc	0.036552	0.19119	0.65	0.69
Residual		0.272997	0.52249		

Number of observations: 15619. Grouping variable: participants, 83

Fixed effects:

	Estimate	Std. Error	t -value
(Intercept)	0.47952	0.01847	25.958
Non-response set	0.03782	0.01612	2.346
Associated color-inc	0.04635	0.01695	2.734
Standard color-inc	0.19640	0.02553	7.694
Associated color-cong	-0.02036	0.01444	-1.410
Standard color-cong	-0.05270	0.01443	-3.651
