# Supplementary material for: A mouse-tracking study of the composite nature of the Stroop effect at the level of response execution
Source: PLoS One. 2023 Jan 19;18(1):e0279036. doi: 10.1371/journal.pone.0279036 (PMC9851562; doi:10.1371/journal.pone.0279036)
Supplement: S3 File — Report the error rates and non responses rates for each experimental condition. (PDF) [file pone.0279036.s003.pdf]

S3. Table of average error rates and time outs (omissions) per conditions

As indicated in the main text, error rate and time out rate (omissions) in the full data set are 0.58% and 0.34% respectively. The Table here below provides those rates per each condition of Stimulus-type variable. To this end, for each participant error and time out rates were calculated in each condition. Then, for each condition, average error and time out rates (and their SD) we calculated across participants.

Stimulus type	Errors (in %)	SD errors (in %)	Time outs (in %)	SD time outs (in %)
Standard colour-congruent	0.1506024	0.8316251	0.1129518	0.5868264
Associated colour-congruent	0.1882530	0.7480599	0.2635542	1.0007642
Standard colour-incongruent	2.5602410	3.5578495	0.6400602	1.6008284
Associated colour-incongruent	0.1882530	0.7480599	0.3765060	1.1338564
Non-response set incongruent	1.7919838	0.2635542	0.8736950	0.4894578
Colour-neutral	0.1506024	0.8316251	0.1506024	0.6733604

Note: Due to the sparsity of the data (most participants make no errors or time outs in some experimental conditions). Therefore some means per conditions across participants (and their SD) are identical.
