# Supplementary material for: A mouse-tracking study of the composite nature of the Stroop effect at the level of response execution
Source: PLoS One. 2023 Jan 19;18(1):e0279036. doi: 10.1371/journal.pone.0279036 (PMC9851562; doi:10.1371/journal.pone.0279036)
Supplement: S4 File — Procedure for clustering the mouse trajectories into partial error clusters and non partial error clusters, and for estimating the partial error rates based on these clustering methods. (PDF) [file pone.0279036.s004.pdf]

## S4. Clustering method for estimating partial error rates

The present section provides more details about our clustering method for estimating partial error rates. Recall that these are aimed to disentangle trials where the cursor is directed towards the centre of the screen (indicating no strong commitment for the incorrect response relative to the correct response) from trials where the cursor is directed more strongly towards the incorrect response (indicating partial errors). Indeed, with mouse deviation measures, these two kinds of trials cannot be properly disentangled in 2-response settings as they both increase mouse deviation compared with more direct responses. Therefore, a method to estimate the partial error rate is proposed here. To this end, a clustering analysis tool implemented in the R package Mousetrap (v3.1.4; [1]) was used for sorting trajectories (based on their dissimilarity) into different types of trajectories.

The data and the code related to this method is provided on the OSF webpage related to this manuscript (https://osf.io/f2cpk/). The OSF webpage contains additional scripts replicating the clustering results with different parameters (i.e., number of clusters above 8).

### *Definition and summary of the method*

Within the Mouse-tracking experimental paradigm, partial errors are defined as response trajectories clearly deviating towards the incorrect response, before being corrected and ending their course at the correct response. Based on this definition, there are two problems with implementations that detect such partial error trajectories based on simple parameters such as the initial direction of the trajectory (e.g., initial angle, initial x coordinate…). First, they impose to set cut-off values, often arbitrarily. For instance, at which point of the trajectory should the initial angle be measured? Over or below which value can an initial trajectory angle be considered sufficient to distinguish between a trajectory initially directed towards the incorrect response vs. towards the display centre. Second, such parameters can miss clear partial errors. For instance, partial errors include trajectories initially directed towards the display centre (or the correct response), then directed almost at the incorrect response

before being corrected and ending at the correct response. Such trajectories would be classified as trajectories deviating towards the display's centre under certain cut-off values on the diagnostic parameter (or correct response).

To address these issues, a data driven approach using clustering analyses is proposed here. It takes into account the whole trajectories instead of parameters evaluated on short parts of the trajectory. These kinds of analyses identify clusters of similar trajectories which correspond to different types of trajectories. They make it possible to identify visually the clusters corresponding to our definition of partial errors and calculate the proportion of trials sorted into these clusters. It is to be noted that Moher and Song [2] used another approach consisting in labelling a trajectory as partial error when it deviates, at any time step (normalized), for more than 1.5 *SD* of the average trajectory of its condition. However, they used a reaching task, in which trajectories are often less complex than in mouse-tracking, with 3 response buttons (one being at the top-centre of the screen), which makes it a different setup.

### ***Estimating mouse trajectories partial error rates using clustering analyses***

#### *Hierarchical cluster analyses*

The mouse trajectory clustering analysis tool implemented in Moustrap R package was used as described in Wulff et al. [3]. First, to ensure that trajectories can be compared properly, the start and end points of the mouse trajectories were aligned (all start and end points at the same coordinate), and that all trajectories with leftward correct response were remapped rightward. Second, for clustering function to estimate the pairwise distance (dissimilarity) of each trajectory, raw trajectories were first represented each with an equal number of points evenly spaced (100 in the present study); then the pairwise Euclidian distance between the trajectories was calculated. From there, two algorithms were proposed to estimate the clusters from the distance matrix: k-means clustering and hierarchical clustering algorithms (HCA). The algorithm and estimation method set by the authors as default was used.

The mt_cluster() function – clustering the input trajectories into a present number of clusters – was used. This number of clusters was initially set to 5 which seemed sufficient to represent the main kinds of trajectories possible with the mouse-tracking setup used in the present study. It should be noted that the analysis was not aimed to search for the best number possible of clusters but for a number of clusters that is sufficient to represent correctly the data. As described later one, such number of clusters was not sufficient and instead, we increased this number to 8 clusters.

For the sake of comparison, a script in the OSF page is provided for reproducing the results from the main text with a 12 clusters analysis (stx_suppl_pe_12clusters.R). The results obtained are exactly the same than by estimating partial errors with 8-clusters analyses. When using HCA, increasing the number of clusters does not result in completely reorganising the clusters but rather divides the existing clusters. This ensures that the classification between partial and non-partial error trials is consistent across clustering analyses with different number of clusters (as long as the total number of clusters allows for discriminating partial from non-partial error trials, see script stx_suppl_CLcomparison.R on the OSF webpage for a demonstration with 8, 12, 15 and 18-clusters analyses).

In the following sections, an indicator (a modified silhouette coefficient) of how well the cluster analysis sorts each trajectory within partial error clusters is also provided (although this is not its main goal).

*Estimating partial errors from 5 clusters analysis*

Figure s1 (top) provides a representation of trajectories sorted by clusters in the 5 clusters HCA. Clusters Cl3 and Cl4 correspond the definition of partial errors outlined above. The former represents trajectories closest to a template where the initial movement reaches the incorrect response and is corrected late in the trial. The later represents trajectories closest to a template where the initial movement is attracted by the incorrect response (although not as much as Cl3). However, the extent to which this cluster includes trajectories simply attracted towards the display's centre is unclear. To

verify this, a modified version of the silhouette coefficient was used. It was tailored to evaluate how close each trajectory in clusters Cl3 and Cl4 is from the other trajectories in PE clusters (Cl3 and Cl4) and in non-PE clusters (Cl1, Cl2, Cl5).

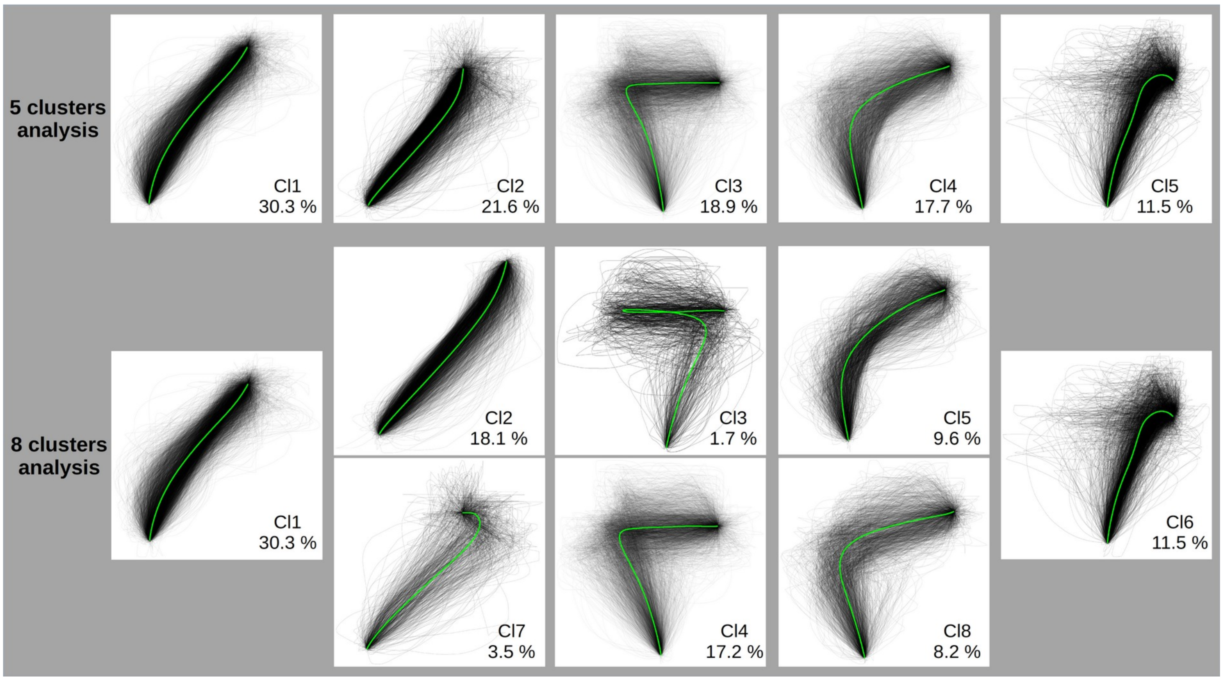


**Figure s1. Distribution of mouse trajectories (for all Stroop conditions) across the clusters estimated through hierarchical cluster analysis with 5 (top) and 8 (bottom) clusters.**
Each cluster is supplemented with its average trajectory (green line). Some clusters of the 8 clusters analysis represent subsets of trajectories from a cluster of the 5 clusters analysis. The spatial layout for the clusters of 8 clusters analysis reflects this subsetting. For instance, Cl3 in the 5 clusters analysis is subsetted into Cl3 and Cl4 in the 8 clusters analysis, all displayed in the same column.

*The silhouette coefficient (an indicator of how well trajectories are sorted)*

Some trajectories are better represented by their clusters than others. Here, the aim is to evaluate whether each single trajectory sorted as partial error (clusters Cl3 or Cl4) is close enough to its actual cluster (or to the other partial error cluster) as compared to other (i.e., non partial-error) clusters. To this end, a silhouette coefficient was modified to reflect the distance of each partial error trajectory to the closest partial error cluster relative to its distance to the closest non partial error cluster. For comparison, the standard version of the silhouette coefficient evaluates how close the trajectories are from the trajectories of their own cluster relative to the trajectories of the closest other cluster. Since trajectories lying between clusters Cl3 and Cl4 can confidently be sorted as partial error, the standard

silhouette coefficient does not address properly the issue of classifying trajectories as partial error or non partial error, motivating us to modify the standard formula.

$S_i$ denotes the silhouette coefficient for mouse trajectory i, where i is a trajectory classified as a partial error (i.e., belonging to a cluster regrouping partial error-like trajectory type). It is calculated as follows:

$$S_i = \frac{D_i^{nc} - D_i^{pc}}{max\left\{D_i^{nc}, D_i^{pc}\right\}}$$

$D^{pc}_i$ denotes the average distance of i to the trajectories of the closest partial error cluster (internal average distance). It is estimated, for each partial error cluster, as average Euclidian distance from i to each trajectory of the cluster such that $D^{pc}_i$ is the minimal average distance across the partial error clusters. While estimated by applying the same procedure, $D^{nc}_i$ denotes on the other hand, the average distance of i to the trajectories of the closest non partial error cluster (external average distance).

The silhouette coefficient ranges between -1 and 1 with positive values indicating a trajectory that is more similar to trajectories sorted in the closest partial error cluster than to trajectories sorted in the closest non-partial error cluster, and negative values indicating the opposite. Its actual magnitude depends on the degree of homogeneity within a given cluster (i.e., small distances between the trajectories sorted in the same cluster).

Figure s2 (left) represents the distribution of modified silhouette coefficients for the trajectories sorted as partial errors (clusters Cl3 and Cl4). The mean silhouette coefficient for partial error trajectories is 0.31 and its median is 0.37. Some trajectories classified as partial error with HCA deviate further from partial error clusters than from non partial-error clusters (silhouette < 0), or are not very distant from a non-PE cluster (low positive silhouette). It is not surprising that silhouette coefficient and HCA do not agree on how to classify certain singular trajectories since distances between the trajectories and the clusters are estimated differently. However, the silhouette coefficient is

a useful tool for investigating how well a cluster analysis with a certain number of clusters satisfy our objective of classifying partial errors.

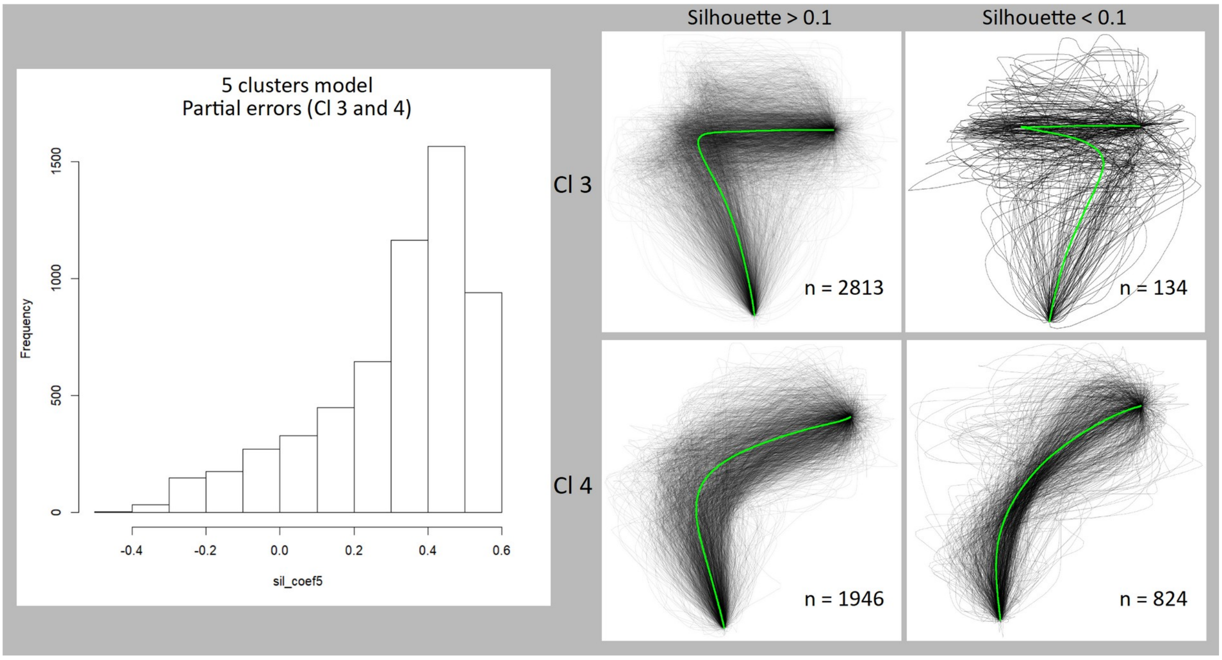


**Figure s2. Left. Distribution of the silhouette coefficients of partial error trajectories for the 5 clusters analysis.**
Right. Mouse trajectories of the partial error clusters (Cl3 and Cl4), with medium/high silhouette coefficients (>0.1) and with low silhouette coefficients (<0.1), n denotes the number of trajectories, green lines denotes the average trajectory.

One way to achieve so is to examine the profile of the trajectories with low (and negative) silhouette coefficient in each partial error cluster, as well as the profile of the trajectories still included in those clusters if low silhouette trajectories are filtered (Figure s2, right). In Figure s2, a cut-off value is set at 0.1 for sorting low silhouette trajectories. On one hand, Cluster Cl4 seems to discriminate poorly between trajectories directed towards the incorrect response and trajectories directed towards the centre. This is evidenced by the significant number of trajectories with low silhouette coefficient (824 out of 2770 trajectories sorted in cluster 4). On the other hand, a much smaller proportion of the trajectories sorted into cluster Cl3 appear to have a low silhouette coefficient (134 out of 2947). As displayed in Figure s2, they include a fair share of trajectories initially directed towards the correct direction, then to the incorrect direction before being finally corrected. It is fair to assume that such trajectories could be classified as partial error as they inform that at some point of the trial, the

incorrect response exerted a sufficient attraction, leading to a clear movement directed to it. If we would use the silhouette coefficient for filtering poorly sorted partial error trajectories, those trajectories would be removed. Therefore, those analyses provide some evidence that five clusters are not sufficient at account for the movement complexity present in our experiment.

*Estimating partial errors from 8 clusters analysis*

In order to select a better suited number of clusters, further HCAs were executed, each time increasing the number of clusters by one, until cluster Cl4 was divided into two clusters, one including trajectories deviating more towards the incorrect response and the other including trajectories deviating towards the display's centre. The HCA with the lowest number of clusters which achieved this was the analysis with 8 clusters.

Figure s1 (bottom) displays the different clusters' trajectory profiles. Three clusters can be identified as partial error: Clusters Cl3, Cl4 and Cl8. Cluster Cl3 represents trajectories initially directed in the correct direction, then directed to the incorrect response before being finally corrected. Cluster Cl4 represents trajectories clearly directed at the incorrect response then corrected. Finally, cluster Cl8 represents trajectories directed towards the incorrect response (but less so than cluster Cl4) before correction. Compared with the 5 clusters analysis, clusters Cl3 and Cl4 are a division of cluster Cl3 in the 5 clusters analysis. Clusters Cl5 and Cl8 are are a division of cluster Cl4 in the 5-cluster analysis, Cl5 being a cluster regrouping trajectories closest to a profile with movement directed towards the centre of the screen, and thus, which cannot be considered a partial error).

Figure s3 displays the results of the silhouette coefficient analysis (left: distribution, right: profiles of the high and low silhouette trajectories). The mean silhouette coefficient of the partial error trajectories distribution is 0.32 and its median is 0.35 which is comparable to the 5 clusters analysis (but we are not certain of the relevance of this comparison). If we set a cut-off value of 0.1 for discriminating low/negative silhouette (trajectories poorly sorted as partial error) and high silhouette coefficients, we count only 2 low silhouette coefficient trajectories (our1of 260 trajectories) for cluster

Cl3, 76 (out of 2687 trajectories) for cluster Cl4 and 463 (out of 1277 trajectories) for cluster Cl8. As illustrated in Figure s3 (right), the profiles of the low silhouette trajectories can still be described as partial error profiles. For cluster Cl8, because of its closeness to the non partial-error cluster Cl5 (movements initially directed to the centre), a fair share of trajectories should lie between those two clusters and classifying them with certainty is impossible.

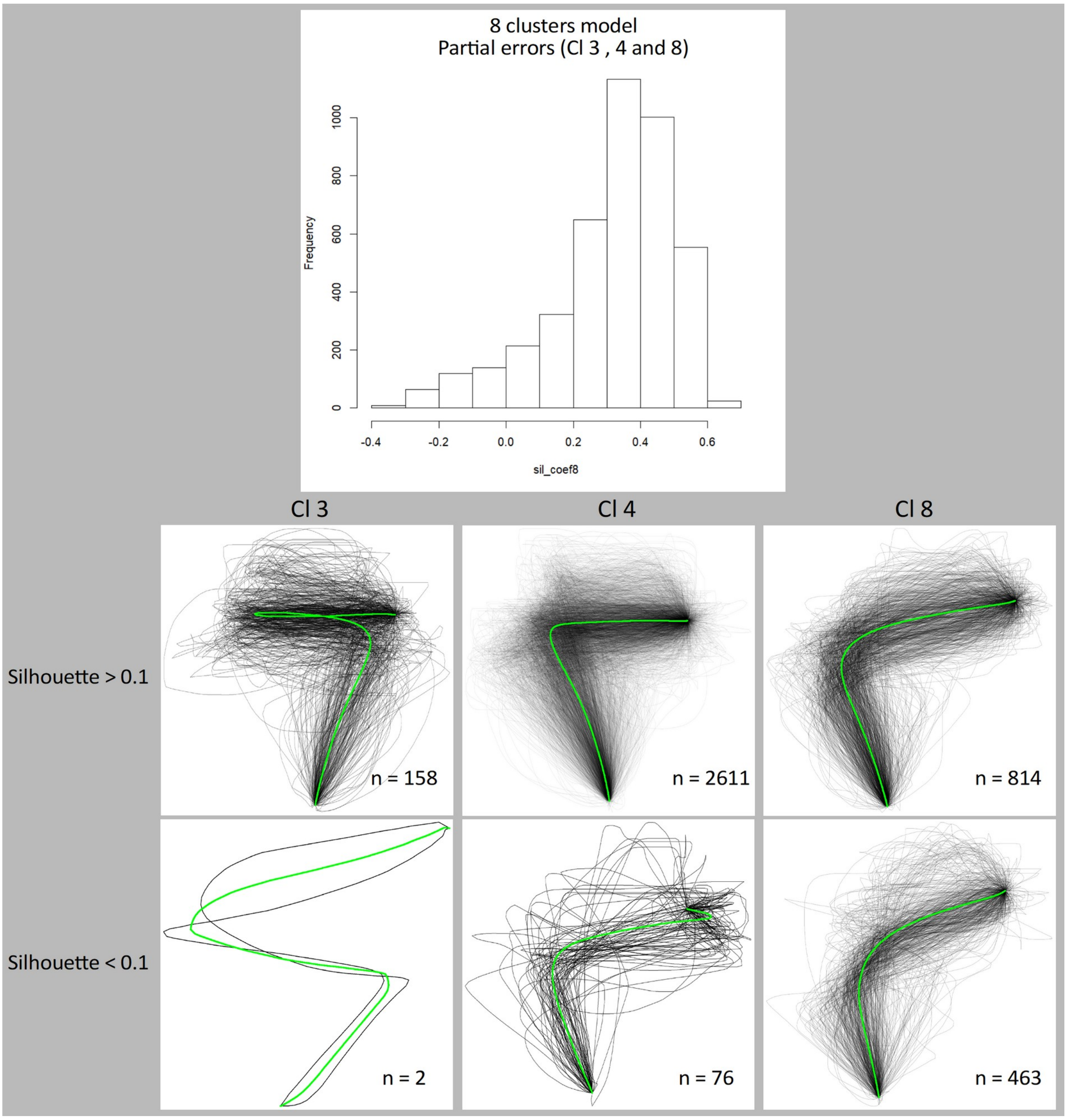


**Figure s3. Top. Distribution of the silhouette coefficients of partial error trajectories for the 8 clusters analysis.**
Bottom. Mouse trajectories of the partial error clusters (Cl3, Cl4 and Cl8), with medium/high silhouette coefficients (>0.1) and with low silhouette coefficients (<0.1), n denotes the number of trajectories, green lines denote the average trajectory.

However, the real purpose of this method is not to classify with confidence each trajectory as partial error (or not). Rather, it approximates an overall estimate of partial error rates per condition, so that the relative differences in error rates across conditions (and thus, the interpretation of the results) do not vary too much depending on the parameters of the clustering procedure (e.g., number of clusters). Additionally, using the term "partial error rates" may be seen as overinterpretation. At very least, this measure can be seen as reflecting how much the incorrect response side of the screen attracts the movement (rather than the centre of the screen). This includes trajectories initially directed towards the incorrect side then corrected, as well as peculiar trajectories reflecting hesitations between responses of different sides (more than one bifurcation on the x-axis).

### *Calculating error rates per participants and conditions and analysing data*

In the present work, trajectories are sorted as as partial error (or not) through the 8 clusters HCA. The silhouette coefficient is only used for exploring our data and estimate the limitation of 5 clusters or 8 clusters analyses. After sorting each trajectory, the error rates per participant and conditions were calculated (so as with an ANOVA, there is one observation per participant and condition).

Since there is only one error rate per condition and participant, the benefice of using LMM over ANOVA is reduced (in this case, these are almost mathematical equivalents). In the present study, LMM was chosen for consistency with the other analyses. Also, one should be very careful when fitting repeated measures ANOVA and estimating marginal means with the emmeans package. In this case, marginal means were biased. Instead, both making paired comparisons with t-tests and using a LMM with emmeans returned the correct means per conditions and differences across conditions. The analyses of partial error rates with ANOVAs + t-tests are provided in Supporting Information S5 (along with those of Response times and Maximal deviation).

### *Parameters of the linear mixed model*

*Random effects:*

| Groups | Name | Variance | Std.Dev. |
|---|---|---|---|
| Participants | (Intercept) | 80.13 | 8.951 |
| Residual | | 80.29 | 8.960 |

Number of observations: 498, grouping variable: participants, 83

*Fixed effects:*

| | Estimate | Std. Error | t-value |
|---|---|---|---|
| (Intercept) | 24.865 | 1.390 | 17.886 |
| Non-response set | 1.240 | 1.391 | 0.892 |
| Associated color-inc | 3.700 | 1.391 | 2.660 |
| Standard color-inc | 11.854 | 1.391 | 8.522 |
| Associated color-cong | -0.823 | 1.391 | -0.592 |
| Standard color-cong | -2.553 | 1.391 | -1.836 |

Note: All SE being identical is to be expected since the random slopes are not estimated (only the random intercept). Therefore, the estimated SE corresponds to the SE of the colour-neutral condition (baseline condition of the dummy variables, i.e. the intercept of the model) and the SE of the other conditions are considered not significantly different.

### ***Discussion***

Due to the novelty of this approach for estimating partial errors rates, further formal tests are required for elaborating an optimal method and validating it. For this purpose, a dedicated study using a simpler conflict task than the Stroop task would probably be beneficial. While the present study could not qualify as a proper validation of the method, the script stx_suppl_pe_12clusters.R (OSF webpage) provides an analysis of the partial errors with a 12 clusters analysis. The results reproduce exactly the results obtained with the 8 clusters analysis. Indeed, as discussed previously, direct correspondence between the clusters of HCA with varying number of clusters (this is also illustrated in Figure s1). Furthermore, script stx_suppl_CLcomparison.R formally verifies this claim with 8, 12, 15 and 18 clusters HCAs. This indicate that the repartition of the trajectories between clusters is constant across clustering analyses with different preset numbers of clusters (sub-clusters in analyses with few clusters form full clusters in analyses with numerous clusters). Provided that the analyses with few clusters still dispatch the trajectories into enough trajectory types without pooling partial errors and non-partial errors together, variations in the number of clusters does not affect the classification of the trajectories

between partial errors and non-partial errors. However, this result should be verified with a dedicated study, with new data. Furthermore, one limitation is that the classification of a cluster as partial or non partial error cluster still carries some subjectivity.

**References**

[1] Kieslich PJ, Henninger F. Mousetrap: An integrated, open-source mouse-tracking package. Behav Res 2017;49:1652–67. https://doi.org/10.3758/s13428-017-0900-z.
[2] Moher J, Song J-H. Context-dependent sequential effects of target selection for action. Journal of Vision 2013;13:10. https://doi.org/10.1167/13.8.10.
[3] Wulff DU, Haslbeck JM, Kieslich PJ, Henninger F, Schulte-Mecklenbeck M. Mouse-tracking: Detecting types in movement trajectories. A Handbook of process tracing methods. Routledge, New York and London: 2019, p. 131–45.
