# Supplementary material for: A mouse-tracking study of the composite nature of the Stroop effect at the level of response execution
Source: PLoS One. 2023 Jan 19;18(1):e0279036. doi: 10.1371/journal.pone.0279036 (PMC9851562; doi:10.1371/journal.pone.0279036)
Supplement: S5 File — Replication of the results with ANOVAs and paired comparisons instead of linear mixed models. (PDF) [file pone.0279036.s005.pdf]

S5. Statistical analyses of summary variables using ANOVAs

Here, LMM approach is supplemented with more standard ANOVAs that estimate the omnibus effect of the Stroop condition. Planned pairwise comparisons using t-tests for estimating the amplitude and p-values of the Stroop components are also provided. It should be noted that LMM-based method presented in the main text and ANOVAs reported here produce very close results. This is because in a balanced factorial design, estimates obtained from simple linear models (such as comparing means in ANOVAs/t-tests, or standard linear regression) and hierarchical linear models/LMMs converge to very similar solutions (if not identical). For instance, the exclusion of a few trials for some participants (time outs, errors) introduces a very slight unbalance in the design (explicitly modelled in LMMs), resulting in small differences in the estimated values. The second difference between the ANOVA/t-tests method and our LMM method is that LMMs generally provide bigger error degrees of freedom due to the fact that they are fitted on individual trials (nested within participants, whose mean intercept and slopes are estimated as part of the fitting procedure). LMM approach provided in the main text estimated those *dfs* using the Satterthwaithe approximation that is mostly influenced by the number participants (levels of the random variable) but still takes into account the number of trials per participants. This results in *dfs* estimated around 90 to 120 (see main manuscript) instead of 82 for a paired samples t-test.

General method

After excluding the erroneous and time out trials the means per conditions and participants (for Response times and Maximal deviation), as well as the percentages of partial errors per conditions and participants were calculated. Then, one-way repeated measures ANOVA with the Stroop conditions (6 levels) as predictor was ran to estimate the omnibus effect of the conditions on each of the three summary measures. Since the sphericity of the residuals was never met, we report the corrected Greenhouse-Gesser p-values (p_{GG}) as well as the amplitude of this correction (ε_{GG} parameter indicating

distance from sphericity; the farther below 1, the more the residuals are non-spherical). For estimating the size of the condition effect (η^2), we used the effect size R package (CRAN, 2022, v. 0.6.0.1). With this framework, the amplitude of each Stroop component is simply estimated by calculating the mean difference between two Stroop conditions which equates in making planned paired comparisons. Bilateral paired samples t-tests was used for doing so. Despite strong hypotheses regarding the direction of the tested differences, the bilateral t-tests was preferred because both R's t.test() function and cohens_d() function (from package effect_size) provide confidence intervals including the two boundaries (lower and upper) only in the case of a bilateral test. Other justifications for this choice is the conservativeness of this setting and the fact that it provides confidence intervals which better match the confidence intervals from our LMMs (which is not surprising since by default, the 99~~5~~% CI estimated from LMMs consider both sides of the t-distribution). Finally, the alpha was set to 0.01 (99% CI, as in the main article) to correct of multiple comparisons (see results section pXX for justification).

~~Finally, t~~Through a power analysis (function pwr.t.test() in R package pwr, CRAN, 2020), we estimated that our sample of 83 participants would allow to detect true effects as small as $d = 0.31$ with a statistical power of 0.8 and an alpha threshold of 0.05, when using bilateral paired samples t-tests at an alpha of 0.05, and $d = 0.38$ at an alpha of 0.01 (correction for multiple comparisons). ~~(function pwr.t.test() in R package pwr, CRAN, 2020).~~

Response times

Means by conditions

(same as Table 1 in the full manuscript)

**Condition**	**mean**	**se**
Standard colour incongruent	1189.39	18.90
Non-response set	1111.25	14.87
Colour-associated incongruent	1074.56	14.15
Colour-neutral	1070.03	12.99
Colour-associated congruent	1044.89	13.29
Standard colour-congruent	1029.27	12.50

Omnibus Stroop condition effect

(repeated measures ANOVA)

	df	ss	ms	*F*	*p*	ε_{GG}	p_{GG}	η^2	η^2_CI
**condition**	5	1379430	275886	81.21	<.001	0.538	<.001	0.137	[0.074, 0.192]
**residual**	410	1392785	3397						

Stroop components

(planned pairwise comparisons with bilateral paired sampled t-test)

Degrees of freedom for all tests = 82, alpha = 0.01.

component	comparison	estimate (*se*) [99% CI]	*t*	*p*	Cohen's *d* [95% CI]
**Stroop effect**	colour incong. - colour congruent	160.12 (12.92) [126.05, 194.19]	12.39	<.001	1.36 [1.07, 1.67]
**Stroop inter.**	colour incong. - colour-neutral	119.36 (13.03) [85.01, 153.71]	9.16	<.001	1.01 [0.74, 1.28]
**Resp. conflict**	colour incong.- non-response set	78.14 (11.30) [48.33, 107.95]	6.91	<.001	0.76 [0.52, 1.01]
**Sem. conflict**	associated incong. - colour-neutral	4.53 (6.78) [-13.35, 22.41]	0.67	0.51	0.07 [-0.14, 0.29]
**Sem relevance**	non-response set - associated incong.	36.69 (6.49) [19.58, 53.8]	5.65	<.001	0.62 [0.39, 0.86]
**Stroop facil.**	colour-neutral - colour congruent	40.76 (6.88) [22.61, 58.91]	5.92	<.001	0.65 [0.41, 0.89]
**Resp. facilitation**	associated congruent - colour congruent	15.61 (6.07) [-0.38, 31.61]	2.57	0.01	0.28 [0.06, 0.50]
**Sem. facilitation**	colour-neutral associated congruent	25.14 (6.04) [9.23, 41.06]	4.17	<.001	0.46 [0.23, 0.69]

Maximal deviation

Means by conditions

(same as Table 1 in the full manuscript)

**Condition**	**mean**	**se**
Standard colour incongruent	0.676	0.029
Non-response set	0.517	0.019
Colour-associated incongruent	0.526	0.021
Colour-neutral	0.479	0.017
Colour-associated congruent	0.459	0.020
Standard colour-congruent	0.427	0.018

Omnibus Stroop condition effect

(repeated measures ANOVA)

	df	ss	ms	F	p	ε_{GG}	p_{GG}	η^2	η^2_CI
**condition**	5	3.181	0.636	42.859	<.001	0.651	<.001	0.148	[0.084,0.205]
**residual**	410	6.086	0.015						

Stroop components

(planned pairwise comparisons with bilateral paired sampled t-test)

Degrees of freedom for all tests = 82, alpha = 0.01.

**component**	**comparison**	**estimate (*se*) [99% CI]**	***t***	***p***	**Cohen's *d* [95% CI]**
**Stroop effect**	colour incong. - colour congruent	0.250 (0.026) [0.180, 0.319]	9.477	<.001	1.040 [0.775, 1.314]
**Stroop inter.**	colour incong. - colour-neutral	0.197 (0.024) [0.133, 0.260]	8.165	<.001	0.896 [0.643, 1.156]
**Resp. conflict**	colour incong.- non-response set	0.159 (0.023) [0.1, 0.219]	7.061	<.001	0.775 [0.531, 1.025]
**Sem. conflict**	associated incong. - colour-neutral	0.046 (0.017) [0.002, 0.091]	2.730	0.008	0.300 [0.079, 0.522]
**Sem relevance**	non-response set - associated incong.	-0.009 (0.016) [-0.051, 0.033]	-0.555	0.580	-0.061 [-0.278, 0.155]
**Stroop facil.**	colour-neutral - colour congruent	0.053 (0.014) [0.015, 0.09]	3.724	<.001	0.409 [0.185, 0.635]
**Resp. facilitation**	associated congruent - colour congruent	0.032 (0.014) [-0.005, 0.07]	2.268	0.026	0.249 [0.030, 0.469]
**Sem. facilitation**	colour-neutral associated congruent	0.020 (0.014) [-0.016, 0.056]	1.499	0.138	0.164 [-0.053, 0.383]

Partial error rates

Recall that partial error rates have been estimated through a cluster analysis with a preset number of 8 clusters, 3 of those being identified as grouping the trajectories closest to a partial error profile.

Means by conditions

(same as Table 1 in the full manuscript)

**Condition**	**mean**	**se**
Standard colour incongruent	36.72	1.73
Non-response set	26.11	1.27
Colour-associated incongruent	28.56	1.50
Colour-neutral	24.86	1.17
Colour-associated congruent	24.04	1.35
Standard colour-congruent	22.31	1.25

Omnibus Stroop condition effect

(repeated measures ANOVA)

	df	ss	ms	*F*	*p*	ε_{GG}	p_{gg}	η^2	η^2_CI
**condition**	5	11032	2206.5	27.48	<.001	0.817	<.001	0.123	[0.062, 0.176]
**residual**	410	32918	80.3						

Stroop components

(planned pairwise comparisons with bilateral paired sampled t-test)

Degrees of freedom for all tests = 82

component	comparison	estimate (*se*) [99% CI]	*t*	*p*	Cohen's *d* [95% CI]
**Stroop effect**	colour incong. - colour congruent	14.41 (1.66) [10.02, 18.8]	8.65	<.001	0.95 [0.69, 1.21]
**Stroop inter.**	colour incong. - colour-neutral	11.85 (1.63) [7.54, 16.16]	7.25	<.001	0.80 [0.55, 1.05]
**Resp. conflict**	colour incong.- non-response set	10.61 (1.44) [6.81, 14.42]	7.36	<.001	0.81 [0.56, 1.06]
**Sem. conflict**	associated incong. - colour-neutral	3.70 (1.47) [-0.17, 7.54]	2.52	0.01	0.28 [0.06, 0.50]
**Sem relevance**	non-response set - associated incong.	-2.46 (1.40) [-6.14, 1.22]	-1.76	0.08	-0.19 [-0.41, 0.02]
**Stroop facil.**	colour-neutral - colour congruent	2.55 (1.18) [-0.55, 5.65]	2.17	0.03	0.24 [0.02 0.46]
**Resp. facilitation**	associated congruent - colour congruent	1.73 (1.10) [-1.16, 4.62]	1.58	0.12	0.17 [-0.04, 0.39]
**Sem. facilitation**	colour-neutral associated congruent	0.82 (1.13) [-2.17, 3.81]	0.73	0.47	0.08 [-0.14, 0.30]

Short concluding comments

The results obtained with ANOVAs are not qualitatively different from the results presented in the main article, obtained by modelling the data with LMMs. The ANOVAs closely approximate the LMM results (or the other way around). Those methods seem to diverge in their approach of data analysis: The ANOVAs are often seen as closer to the real data (not transformed through a modelling procedure) and one tends to see them as a data analysis method more than a statistical data modelling method. But it is important to point out that even an ANOVA + planned paired comparisons method incorporates some form of statistical modelling (maybe more implicitly). The process of averaging is a case of parameter estimation using squared error minimization and therefore, it does not conceptually

diverge too much from our LMM method consisting in explicitly modelling the data and estimating the marginal means and differences across means (marginal effects) from the predicted values of the model. Moreover, squared error minimization and log-likelihood maximization (used in LMMs) converge towards similar solutions in linear models (such as ANOVAs and LMMs), with balanced designs. However, looking at the exact parameter values and their confidence intervals, one can notice that small effects (such as facilitation effects or semantic interference) generally have larger confidence intervals with LMMs, despite a larger degree of freedom than with ANOVAs. This may indicate that LMMs may be slightly more conservative, probably because of their better account of the error in the data.
