# Supplementary material for: A mouse-tracking study of the composite nature of the Stroop effect at the level of response execution
Source: PLoS One. 2023 Jan 19;18(1):e0279036. doi: 10.1371/journal.pone.0279036 (PMC9851562; doi:10.1371/journal.pone.0279036)
Supplement: S6 File — An evaluation of the individual differences in the Stroop components as well as a split-half reliability test of the within-participant variability. Unfortunately, the present study not being tailored for it those analyses are underpowered. (PDF) [file pone.0279036.s006.pdf]

S6. Analysis of within and between-participants variability

In this section, we present plots depicting the variability of the Stroop components. The first section focuses on between-participant variability. The second section presents a split-half reliability test quantifying within-participant variability. Since the current study was not designed to formally study the variability of the Stroop components, we also discuss the limitations of this analyses of our dataset. To foreshadow those limitations, such studies require a larger number of trials per condition than included. Still this Supporting Information section provides a crude idea of the between- and within-participant variability in our study.

Between-participants variability (individual differences)

The objective of this section is to estimate the magnitude and 95% confidence interval of each Stroop component for each participant, in order to get a visual representation of the individual differences in our experiment.

Method

While between-participants variability analyses often make use of hierarchical/mixed-effects regressions including participants as a random effects variable, it was not possible to use such models in the present work. Indeed, in order to estimate the Stroop components (marginal effects) properly with an LMM approach, a model with a full random structure, or at least a model where all random slopes (slopes' variances) are estimated, would be needed. This was not possible given that such models led to overfitted (or non-converging) models with the current experiment's data, which led to biased estimates of the random effects. This is a direct consequence of a lack of sufficient trials per

participant and condition for the purpose of estimating individual differences. In particular, increasing this number often resolves overfitting problems in linear mixed models [1].

Instead, for each participant, a standard paired-comparison analysis was conducted using the t.test() R function to test the differences across stimulus types. This function permits the extraction of both the differences across stimulus types (i.e., Stroop components) and the 95% confidence interval (CI) of the difference (based on the t-distribution). Since partial error rates are expressed as percentages, it was not possible to get reliable CIs with this function. Instead, the 95% CI of the difference between percentages was calculated with the following formula (in pseudo-code):

```
CI(d, 95%) = d +/- z0.95*sqrt(p1*(1-p1)/N1 + p2*(1-p2)/N2)
```

Where: CI(d, 95%) is the 95% confidence interval for the difference d (in percentage) between two stimulus types (Stroop component), p_1 and p_2 are the partial error rates of each stimulus type (with $d = p_1 – p_2$), N_1 and N_2 their respective trial counts and $z_{0.95}$ is the z-critical value for a confidence level of 95% (i.e., $z_{0.95}$ ~1.96). Finally, sqrt() is the square root function.

Figures s4, s5 and s6 (here below) present the magnitude of each component and their 95% CI, per participant, for each of the summary measures (namely, response times, maximum deviation and partial error rates). The observation count per stimulus type (and participant) did not exceed 32 trials and therefore, the estimates per participant are not powerful enough. Besides, being at the trial level, within-participant variability is not controlled through averaging like it is in analyses made at the level of the participants, where trials are aggregated per condition and participant. This results in noisier estimates and reduces the statistical power further [2]. Given that the current experiment was not tailored for reliable individual differences estimation, only the signs of the estimates are highlighted (although the CIs are also displayed to provide an appreciation of the uncertainty around the component's value). Positive components are displayed in green and negative components in blue.

Response times (RT)

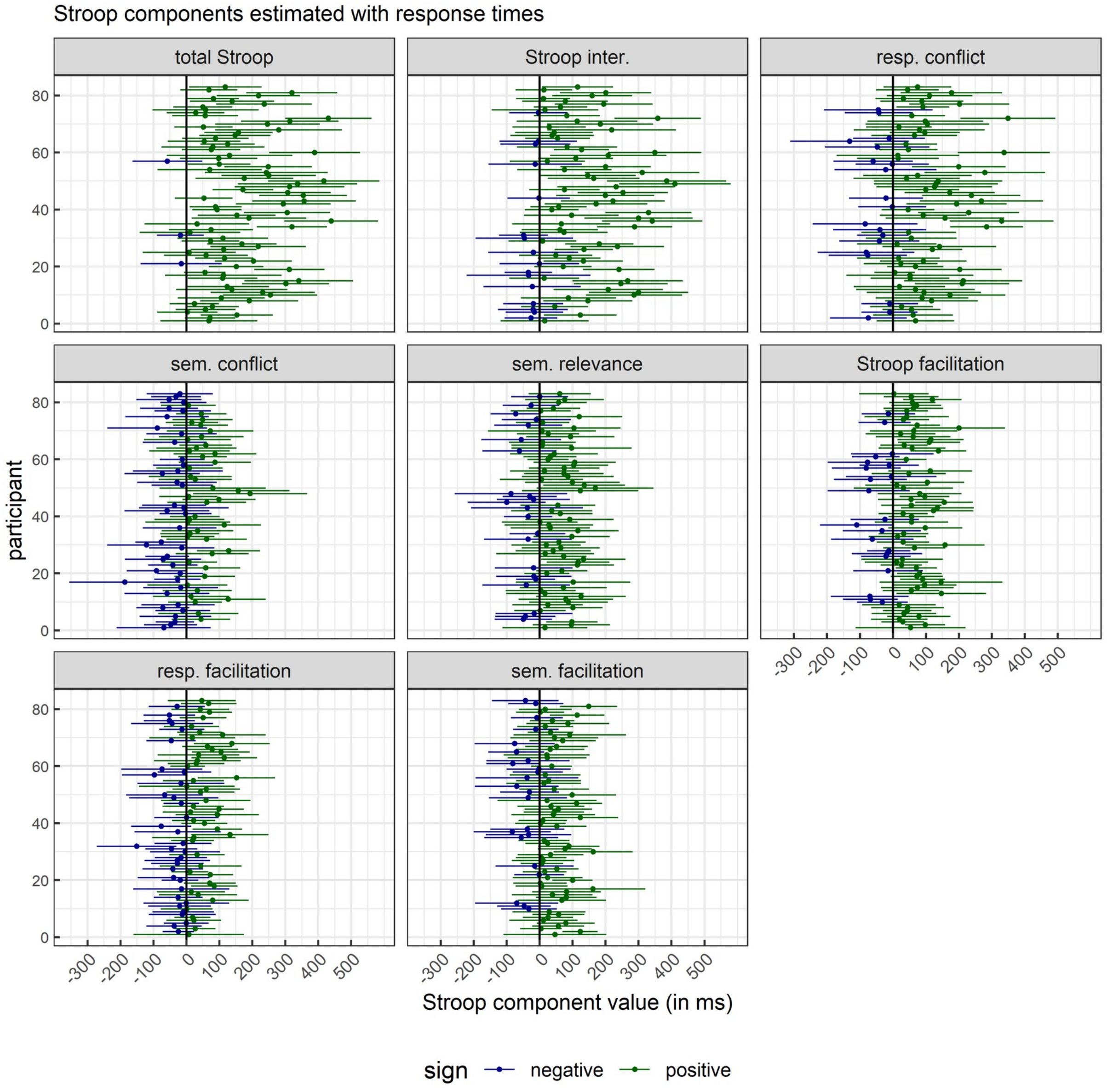

Figure s4. Stroop components estimated with Response times for each participant
Dots represent the average components' amplitude, error bars correspond to their 95% confidence intervals. Green: positive component, Blue: negative component. Components are estimated through averaging stimuli types within participants and contrasting them.

Maximum Deviation (MD)

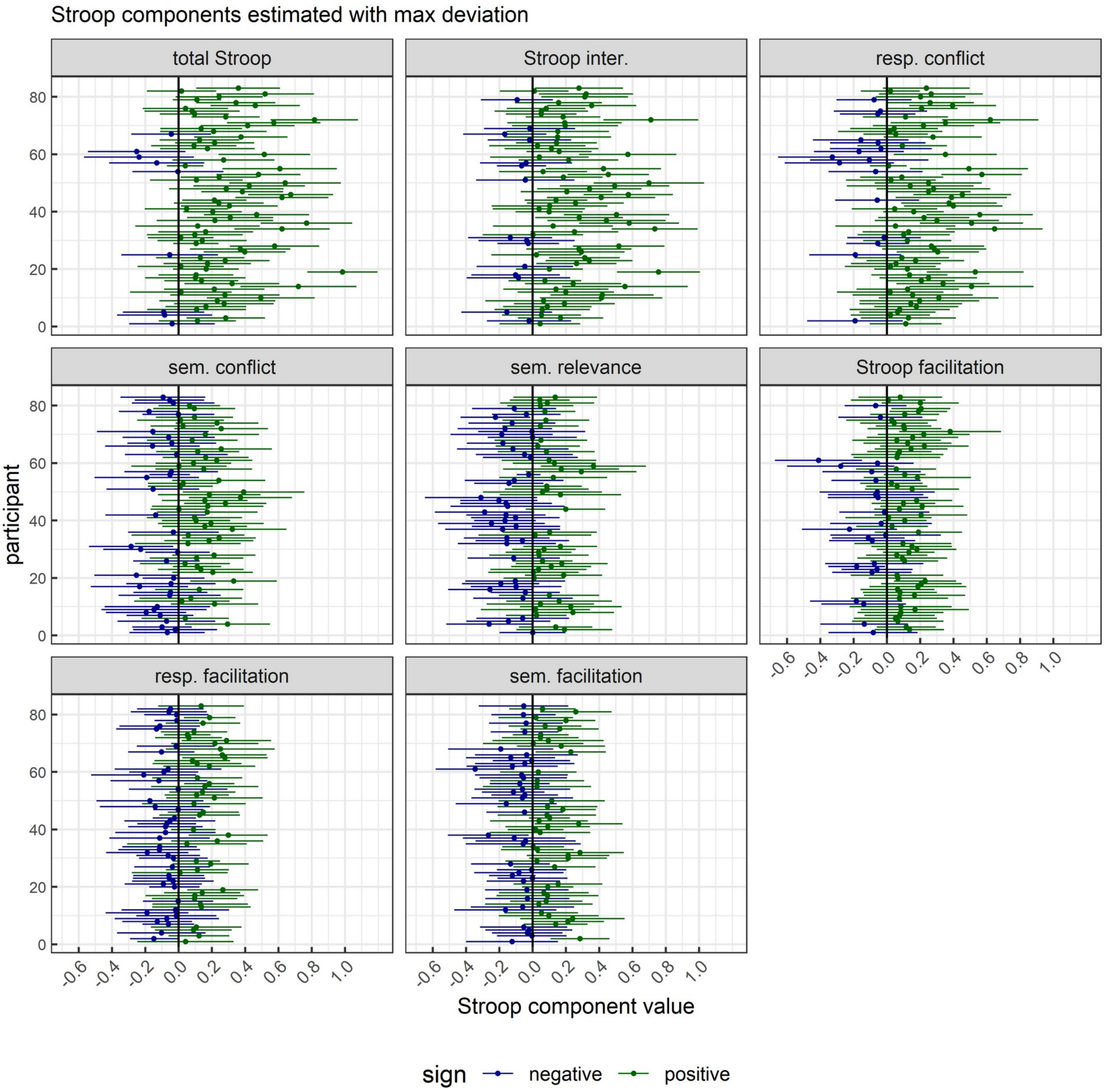

Figure s5. Stroop components estimated with Maximum deviation for each participant
Dots represent the average components' amplitude, error bars correspond to their 95% confidence intervals. Green: positive component, Blue: negative component. Components are estimated through averaging stimuli types within participants and contrasting them.

Partial error rates (PE)

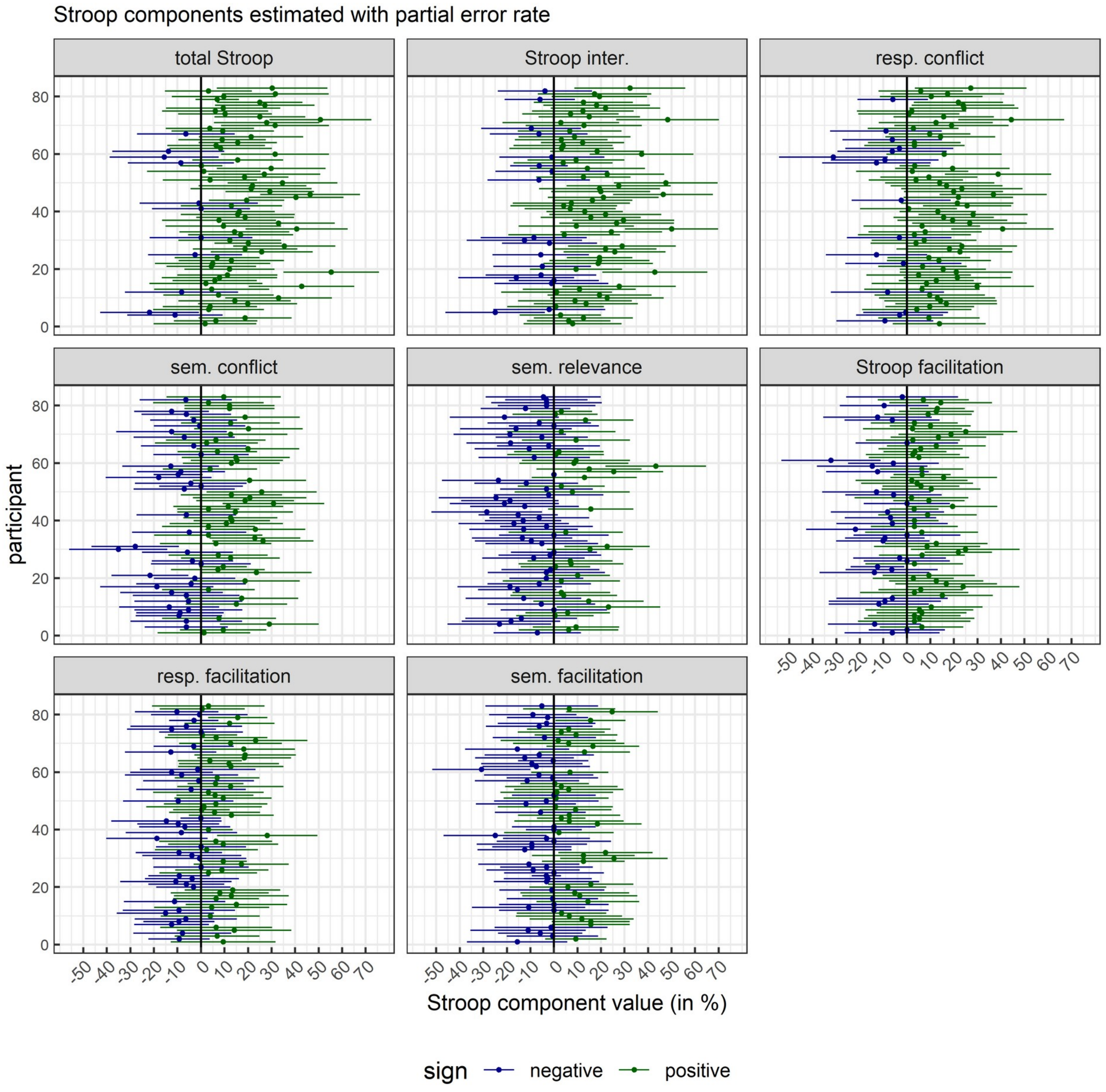

Figure s6. Stroop components estimated with Partial error rates for each participant
Dots represent the average components' amplitude, error bars correspond to their 95% confidence intervals. Green: positive component, Blue: negative component. Components are estimated through averaging stimuli types within participants and contrasting them.

Discussion

Overall, the components with the largest amplitude (i.e., total Stroop effect, Interference effect and Response conflict) have estimates with a consistent, positive sign across participants. This consistency drops with the amplitude of the components, although for the components with a 99% CI

at the group level (see results section in the full article, or S5) which excludes 0 (i.e., Semantic conflict for MD and PE, semantic relevance for RT and Stroop facilitation for MD and RT), the majority of the participants (broadly 60-75%) had a component sign consistent with the group effect. Despite this consistency in sign, sizeable variations can be observed in the amplitude of those components across participants and the 95% confidence intervals provide a broad estimation of the uncertainty around those estimates. Indeed, as highlighted in the Method section, those analyses lack statistical power (max 32 observations per condition and no control of within-participant variability by averaging or via a linear mixed model) and cannot reliably estimate the components at the individual (participant) level. Therefore, future studies investigating individual differences in the Stroop task should aim for a much larger number of trials per condition (for each participant), especially since, compared with studies aiming to estimate Stroop components at the group level, within participant variability cannot be controlled through averaging trials with same stimulus type. Finally, a large number of trials per condition/participant also allows the estimation of individual differences through a linear mixed model approach.

Within participants

The objective of this section is to quantify the within-participant reliability of the Stroop components using a split-half reliability test consisting in calculating the correlation between the Stroop components estimated on each half of the trials for each participant. The higher the correlation, the lower the within-participant variability (the component estimated on one half is highly predicted by the component estimated on the other half of the trials).

Method

Given that only 32 trials per participant and stimulus type were available, a resampling method was used to split the trials in half. At each iteration, the trials of each participant and stimulus type are randomly split in two halves, ensuring that the number of trials per stimulus type and participant are approximately constant across halves. Then, the components are estimated for each half (two estimates per participant and Stroop components) and the correlation across participant is estimated. We repeat this procedure through 1000 iterations, each re-sampling a different random split (to ensure reproducibility, random seeds used at each iteration were generated with a master random seed of 198). Finally, we estimate the split-half correlation for each component by retrieving the 2.5th, 50th and 97.5th percentiles of the resulting distribution of 1000 resampled correlations. The 50th percentile corresponds to the median correlation (the split-half correlation estimate), and the lower and upper percentiles correspond to the boundaries of the 95% CI of the sampled split-half correlations.

Fig s7, s8 and s9 (there below) display the split-half correlation (reliability) of each Stroop component, estimated with the summary measures (RT, MD and PE) with their 95% CI (estimated with the percentiles of the resampled distribution as explained above).

Response times

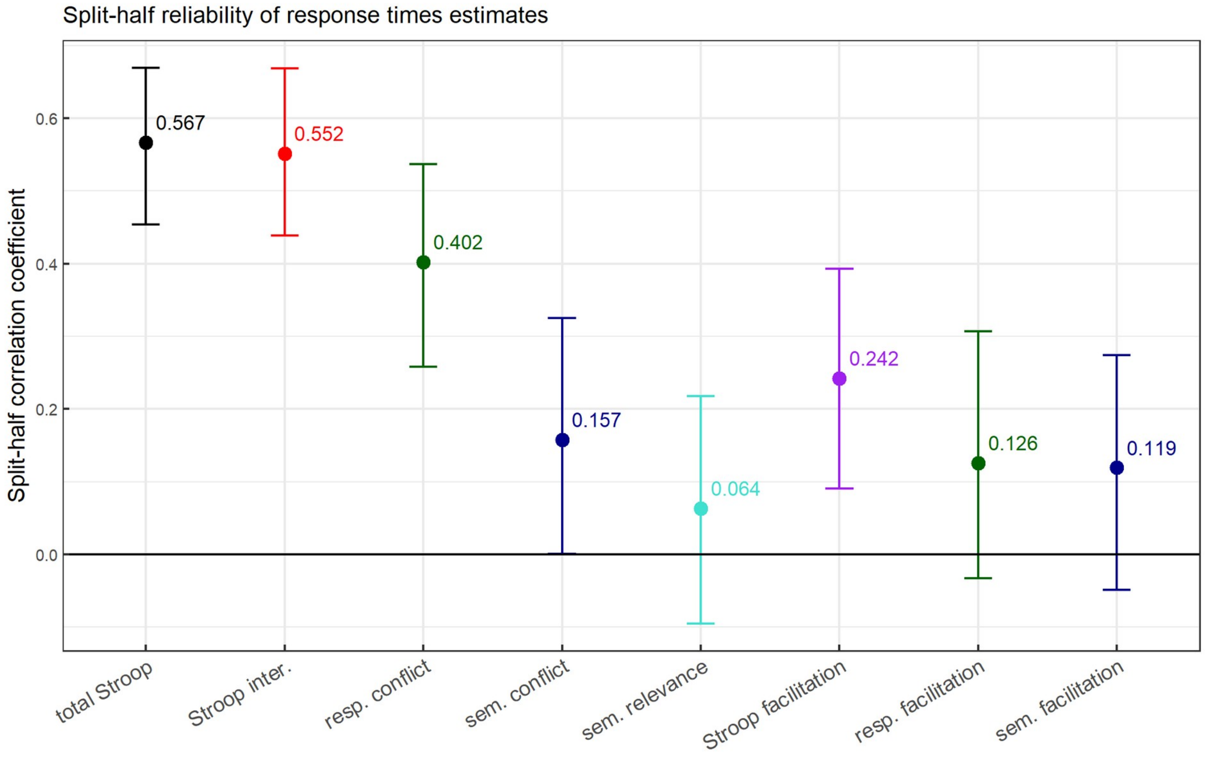

Figure s7. Split-half reliability (Pearson correlation) per Stroop component estimated with Response times

Maximum deviation

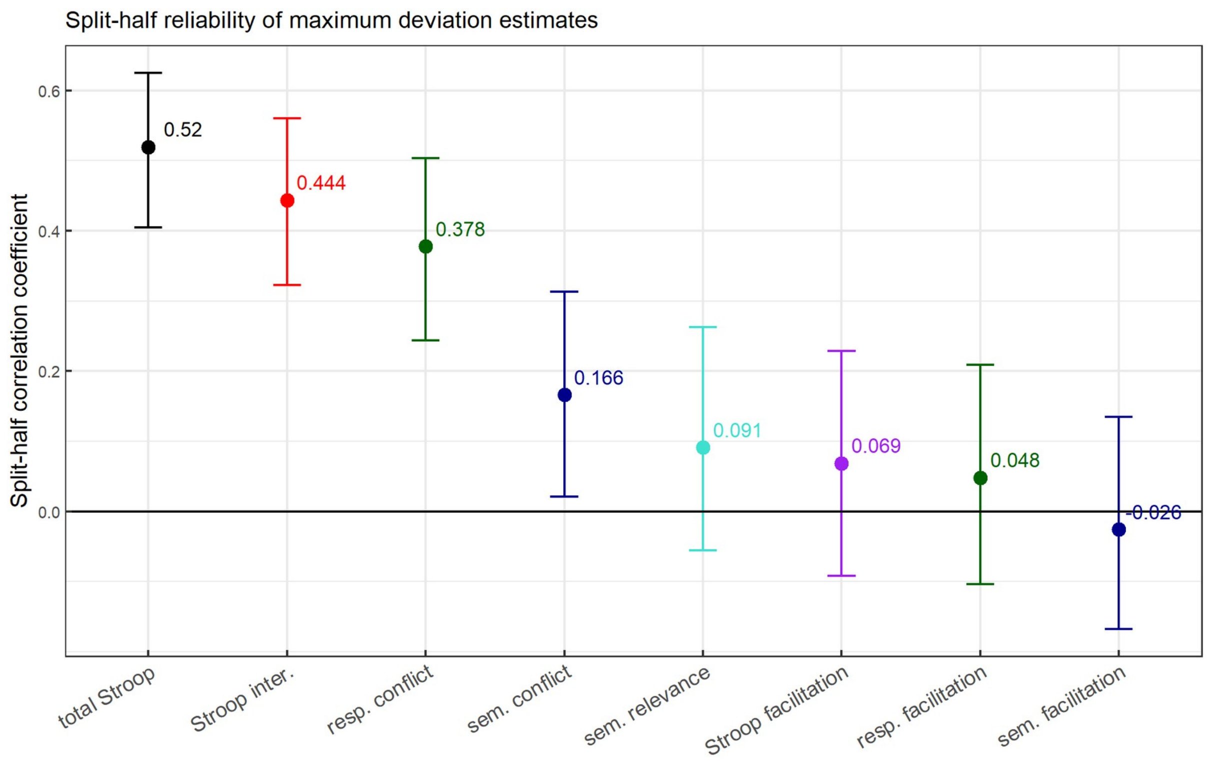

Figure s8. Split-half reliability (Pearson correlation) per Stroop component estimated with Maximum deviation

Partial error rates

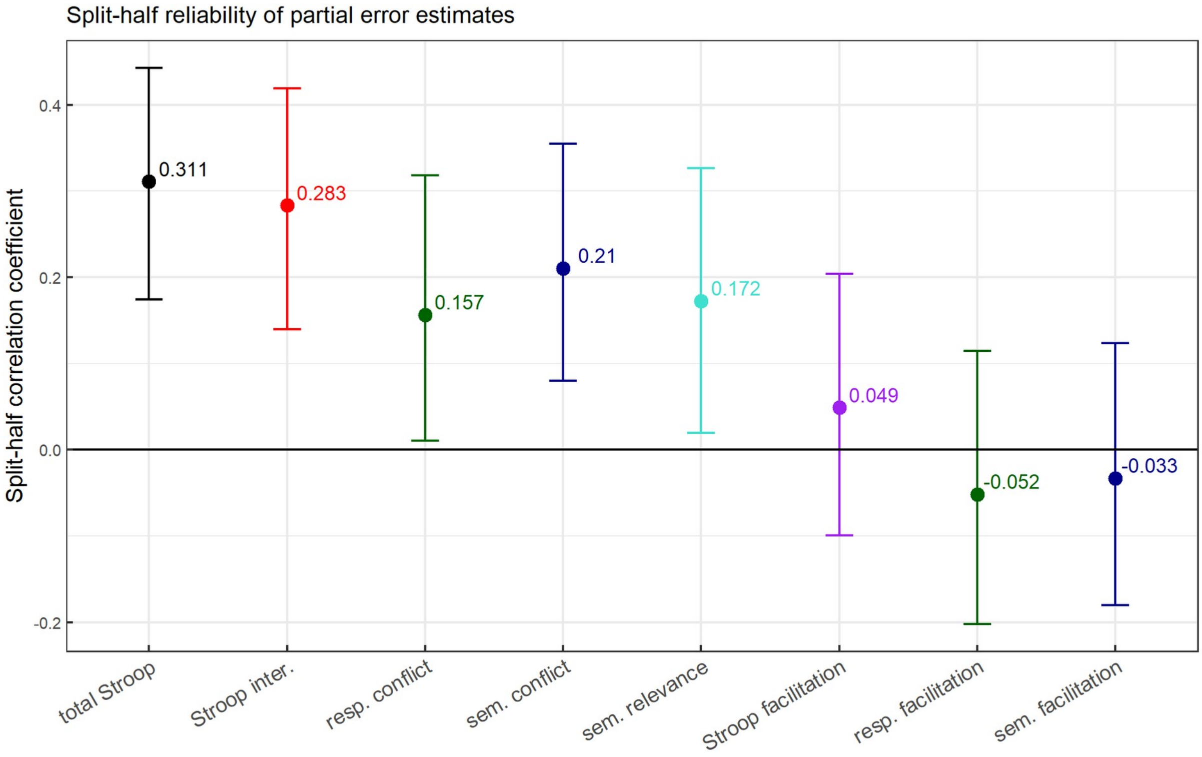

Figure s9. Split-half reliability (Pearson correlation) per Stroop component estimated with Partial error rate

Discussion

Overall, the analyses indicate fairly high within-participant variability. At best, the median correlation (over the resampled correlation distribution) between the two halves of the trials reaches 0.52 (~27% of common variance) and 0.58 (~33% of common variance) for the Stroop effect for MD and RT respectively. The lower magnitude components in our experiment barely achieved a correlation of 0.2 (~4% of common variance). While this seems poor, elevated within-participant variability is to be expected in mouse tracking, given that movement durations span over a longer time than with reaction time (button pressing tasks) – i.e., up to 2500 ms in the present experiment) – and mouse trajectories can vary a lot (see Figure 3 in the main article). This is probably the main reason why standard analysis methods in mouse-tracking [3] rely on aggregating/averaging trajectories per

conditions and participant to control within-participant variability (but see recently proposed clustering analyses for analysing different types of trajectories [4], which we use in the present study for estimating PEs). Importantly, such averaging was very weak in the analyses presented above since for each half, only a maximum of 16 trials were aggregated to estimate the components. This also explains why the correlation for the Stroop components estimated with PE are generally lower than those estimated with RT and MD, since they are based on percentages calculated on 16 observations.

Therefore, as for studying individual differences (and quantifying between-participants variability), studying within-participants variability requires designs tailored for this purpose, therefore including many more trials per participant and condition. Further studies are needed to quantify this variability. For this purpose, psychophysical methods – which favour a very high amount of trials for the purpose of controlling within-participant variability, over participant sample size, often hundreds of trials per condition, – provide interesting tools [5] but using mixed designs with a reasonable number of participants and a high number of trials per condition (probably at least triple the number of trials included in the present experiment, i.e., over ~100) would also be suitable.

References

[1] Bates D, Kliegl R, Vasishth S, Baayen H. Parsimonious mixed models. ArXiv; 2018. https://doi.org/10.48550/arXiv.1506.04967.

[2] Baker DH, Vilidaite G, Lygo FA, Smith AK, Flack TR, Gouws AD, Andrews TJ. Power contours: optimising sample size and precision in experimental psychology and human neuroscience. Psychol. Methods 2020;26:295–314. https://doi.org/10.1037/met0000337

[3] Hehman E, Stolier RM, Freeman JB. Advanced mouse-tracking analytic techniques for enhancing psychological science. Group Process Intergroup Relat 2015;18:384–401. https://doi.org/10.1177/1368430214538325.

[4] Wulff DU, Haslbeck JM, Kieslich PJ, Henninger F, Schulte-Mecklenbeck M. Mouse-tracking: Detecting types in movement trajectories. Handb. Process Tracing Methods. Routledge, New York and London: 2019, p. 131–45.

[5] Smith PL, Little DR. Small is beautiful: In defense of the small-N design. Psychon Bull Rev 2018;25:2083–2101. https://doi.org/10.3758/s13423-018-1451-8
