# Supplementary material for: A mouse-tracking study of the composite nature of the Stroop effect at the level of response execution
Source: PLoS One. 2023 Jan 19;18(1):e0279036. doi: 10.1371/journal.pone.0279036 (PMC9851562; doi:10.1371/journal.pone.0279036)
Supplement: Attachment — Submitted filename: renamed_e1b6d.docx [file pone.0279036.s007.docx]

Reviewers' comments:

5. Review Comments to the Author

Please use the space provided to explain your answers to the questions above. You may also include additional comments for the author, including concerns about dual publication, research ethics, or publication ethics. (Please upload your review as an attachment if it exceeds 20,000 characters)

Reviewer #1: The present study addresses the dissociation of semantic/response conflicts of Stroop effects. Replacing the conventional reaction-time paradigm, the authors use the mouse-tracking technique to assess the conflict/facilitation effects at both decision and execution levels. The experimental design is well-thought-out; the results, particularly the analyses of partial errors, are inspiring. Meanwhile, some limitations of the current research have been discussed.

Thanks very much to Reviewer 1 for his very useful feedbacks. We tried to respond to those as best as possible and modify the MS accordingly. The specific pages referencing the changes are based on the version of the MS with highlighted modifications.

Before this paper is suitable for publication, several issues need to be addressed.

1. One of the main claims in this paper is that mouse-tracking helps reveal the potential overlap between semantic decision and response execution of Stroop effects, as shown by features of mouse trajectories such as partial errors. However, given the nature of the reaching task per se, there are always more or less partial errors observed, since reversing the direction halfway during reaching is often seen in mouse-tracking studies. Thus, it seems farfetched to interpret the partial errors as evidence to overlap of cognitive processes in a specific task (i.e., Stroop) in the current study. Even if previous research with different paradigms suggests that the overlap could exist, it is unclear whether the partial errors in the current study stem from the overlap of selection/execution or other underlying cognitive processes. The authors may want to discuss this limitation further and be more careful about the conclusion.

Thanks very much for this interesting comment. There are actually two separate issues that currently dominant single-stage response models of the Stroop effects are unable to account for:

1) As discussed p.4-6 (first introduction section), these models assume that response conflict (i.e., a unique conflict driving the Stroop interference effect) is assumed to be resolved at the level of response selection. However, only a handful of studies have addressed this assumption of no conflict at the execution level – shared by these extant theories of the Stroop effect – directly and this with actually relatively mixed results (see previously referenced for discussion). Therefore, more studies are needed and mouse-tracking can address this issue of a potential overlap between response selection and execution more directly. Here, partial errors are helpful because – as R1 knows – mouse deviations do not necessarily reflect movements toward an incorrect response. Said differently, they do not necessarily speak to processes involved in the selection of a response (and to its overlap with the execution) as directly as PE do. While R1 is right that hand-tracking induces PE by design (the new version of the Discussion sections makes this issue explicit), the differences between conditions are inconsistent with the idea that this simple explanation can account for all the results reported in the present study. We still hope that we adequately addressed R1’s concern in our new version of the Discussion section (see p.28-31, first discussion section).
2) The aforementioned models assume in parallel to the issue 1, that all conflict there is in the Stroop – is response conflict. Accordingly, as discussed in the Discussion section, these models assume that the interference produced by semantic associates or out of the set items is response conflict. The multiple models (at least the one of Zhang and colleagues) on the other hand assume that an additional type of conflict occurs at the stimulus level – conflict that is semantic in its nature (i.e., conflict that occurs in an amodal semantic network, see p.6-7, second introduction section). While any conflict items should produce mouse-deviations (or slow processes involved in the selection of a response), partial errors are more likely to occur for items involving response conflict specifically (i.e., standard color-incongruent items). Indeed, these latter items should lead to the co-activation of incorrect and color response – co-activation that should be seen in a greater number of partial errors. We hope that our additions to both Introduction section and slight changes in the Discussion section (p.32-36, second discussion section) now clarify the issue raised by R1 about how mouse-tracking and namely PE speak to qualitative difference between semantic and response conflict.

Finally, more generally about how partial errors (in mouse-tracking tasks for the case of the current study, but also in button-pressing studies) provide direct evidence that response selection and execution overlap:

Partial errors occur when a motor signal towards the incorrect response is sent to the effectors (and measured at the level of the effectors), but is inhibited/corrected before the response is completed. For such correction to occur, the response selection must continue after movement initiation (or while EMG signals are recorded at the level of the effectors in button pressing tasks). This is the reason why we use the partial error rate as indicative of an overlap between response selection and execution. As indicated in the MS, in Mouse-tracking, they are characterized with movements clearly deviating towards the incorrect response but corrected later on implying a certain level of overlap (although some movements deviated towards the center may also be partial error, but we cannot be certain of it and thus our clustering procedure aims to exclude them from the partial error rate). The MS now shortly reminds this definition in the discussion p.30 and the introduction of the first discussion section p.29 emphasize this idea.

2. For those Stroop effects examined, the authors report the 95% CI, which corresponds to a p-value criterion of 0.05. However, since there are multiple comparisons conducted, the risk of false-positive is above 5% when 95% CI is repeatedly applied. Thus, p-value corrections for multi-comparisons are needed. Please provide details about p-value corrections, if any. In addition, reporting only 95% CIs is insufficient for readers to evaluate those effects; effect sizes should also be included.

Thank you for this important comment.

We reran the analyses of the summary statistics (Response time, Maximal deviation and Partial error rates) with a more conservative criterion (alpha = 0.01; 99% CIs) and used those analyses for the MS. Not being able to implement the Holm correction given our methodology, we chose such alpha because it corresponds to a Bonferroni correction with 5 comparisons. While in the study, we make 8 comparisons, 3 of them (total Stroop, interference and facilitation) encompass the 5 lower order comparisons (the interference and facilitation components), see Fig 1. In our study, 3 comparisons always resist to a Bonferroni correction with 8 comparisons (alpha = 0.05/8), i.e., total Stroop, Interference and Response conflict. Therefore, compared with Holm correction, using a global alpha of 0.01 leads to identical decisions for the 4 comparisons with the smallest p-value (despite an equal or smaller alpha than 0.01 with Holm) and more conservative decisions for the 4 comparisons with the biggest p-value (using Holm: alpha > 0.01).

We now mention the use of alpha = 0.01 at p.18 of the MS, with justification.

However, we are not able to provide proper effect sizes for the analyses in the MS. While the emmeans R Package we use includes a function for calculating effect sizes on marginal effects, it requires an error estimate. But LMMs estimate several error parameters (residual, random intercept, random slopes) and determining which ones to include in the effect size calculation is not straightforward. Indeed, to our knowledge, this is still a debated question in the literature about LMMs and currently, we do not know how to provide such effect sizes. However, we can add them in the MS if Reviewer 1 knows about a solution to this problem.

Although this is not a proper effect size calculation, in the discussion, we compare the magnitude of the Stroop components in our study, with their magnitude in previous study (obtaining comparable magnitudes). Furthermore, effect sizes are provided for our ANOVA+paired comparisons analyses in Supporting Information S5, and referred to p.18 of the MS.

3. In mouse-tracking analysis, there are alternative features of mouse trajectories to assess the deviation. For example, AUC (area-under-the-curve) is another often-used measure for deviation, especially in semantic conflicts (e.g., Xiao & Yamauchi, 2015). Given that the MD and AUC are usually positively correlated, why choose MD over other features (e.g., AUC) as the index of semantic conflicts? The authors may consider reports other relevant measures as well or explain the reason for relying on MD.

MD and AUC are close measures (highly correlated, mean correlation across participants ~0.85), as pointed out by Reviewer 1. We chose MD for a convenience reason and for a more theoretical reason.

Convenience reason: MD is both more consistent (mathematically, i.e., the metric is an Euclidian distance) with the way we analysed the deviation across time steps, and it is not directly covered in those analyses since maximal deviations occur at different time steps from one trial to another, i.e., deviations occurring at different (normalized) time steps are compared with MD, but not with deviation per (normalized) time steps. On the other hand, AUC being a global measure of the deviation over the whole trajectory, it provides very similar information than the deviation by time step analyses. To us, MD overlaps less with time-based deviation analyses than AUC, and is based on the same metric (Euclidian distance/ orthogonal projection on the ideal trajectory).

Theoretical reason: While very close and related measures, we believe AUC and MD conceptually differ in the way they represent movement deviation. AUC being a general estimation of the deviation along the whole trajectory, it originally aims at quantifying a gradation of the mouse deviation that is thought to reflect how the presented stimulus relates with both responses (see the seminal papers by Spivey and colleagues, 2005-2009, following the continuous mind hypothesis). On the other hand, MD estimates the (maximal) commitment for the incorrect response leading to a movement in that direction with the underlying idea that a strong commitment leads to a movement (almost) reaching the incorrect response. While both measures are strongly related, some trajectories can have a large MD and moderate AUC, for instance when it is characterized by a first movement broadly directed to the correct response, followed by a sharp movement to the incorrect response (clear decision) followed by a (sharp) correction: In this case, the AUC underestimates the commitment to the incorrect response which occurred at some point in the movement. Large AUC with moderate MD corresponds generally to trajectories mostly directed towards the centre before being directed towards the correct response, which is problematic in our experiment because of the movement initiation deadline encouraging such behaviour. Furthermore, the Stroop effect resulting from the integration of different sources of information which can be clearly distinguished (e.g., ink colour and written word), it may lead to complex interactions between the decision process and the response movement. By comparison, Spivey and colleagues generally proposed decisions based one source of ambiguous information (or several indistinguishable sources), more suited to evaluate a gradation. Our intuition there was that in the case of our experiment, the MD may be a better “general purpose” deviation metric with regard to this complexity (although not perfect either).

Currently, we do not think that AUC measures are necessary for the current study. However, if Reviewer 1 think this is necessary, we can provide those analyses in the supporting material (we would prefer this solution since the article is already quite long).

4. In lines 204-205, “participants were also asked to identify the font colour of associated (e.g., SKYgreen) non-response set incongruent trials…” How exactly were participants instructed to do with “non-response?” Please clarify. It may cause confusion because some readers may interpret “non-response trials” as being “completely ignored/no response at all.”

As mentioned on p.9, for all items, the participants were instructed to identify the ink colour as quickly and accurately as possible. Said differently, responses are given for all items. The labels used for different items (see now newly introduced Figure 1) are the terms used in the Stroop literature but these labels (including the “non response” that leaded R1 to possibly imagine that the response is withhold) are never mentioned to them. Participants still have to indicate the colour with which those items are written among those four ink colors. Still, we hope that our polishing of the Present study section improved the clarity of our message.

5. Make sure the first line of each paragraph is indented in a consistent manner.

Done, thank you.

Reviewer #2: Classically, delay in response observed in Stroop tasks has been attributed to the conflict in the input processing stage. In this paper, using a mouse tracking task instead of a simple button press, the authors aimed to decompose the different types of conflict processing in the Stroop task which evolves in the response execution phase.
Response times, maximum deviation towards the interfering targets and the partial error estimate were extracted from the cursor trajectory during the response execution. Authors evaluated the Stroop interference effect and facilitation effect using these measures, decomposing them into response conflict/facilitation and the semantic conflict/facilitation components by setting appropriate control conditions. Authors successfully showed the different magnitude of effect induced by these different factors.
The experiment is performed thoroughly, and the manuscript is written clearly. I believe this shows an elegant way to differentiate the factors contributing to the classical Stroop effect, particularly in the response execution phase.

We would like to thank Reviewer 2 for his interesting feedbacks. We tried to provide acceptable responses and to modify the MS when necessary. The specific pages referencing the changes are based on the version of the MS with highlighted modifications.

Below are the points which need more clarification.

1) One of the main messages of this paper is that the stroop interference effect is not necessarily solved before the action execution but can evolve during the response execution phase. I do understand that the participants are purposefully instructed to initiate the action quickly to facilitate the decision to overlap with the execution. However, no difference in initiation times between different conditions seems still very strange to me (167ms-170ms). It seems like participants are performing “Go before knowing the goal” type task (Chapman et al., 2010). Please discuss the reason for why there is no difference in the initiation times, and what can be affected by this.

Thank you for this interesting observation. We agree with the “Go before knowing the goal” interpretation that is now shortly discussed in the discussion p. 31. Thank you for providing the relevant reference.

2) If I am understanding correctly, if the velocity is similar, response time and the maximum deviation should correlate. How exactly do they correlate, or do they not? In some cases (semantic conflict), authors found dissociation between the response time and the maximum deviation measures, however, I was wondering what this exactly means; is it simply indicating the difference in the noise level between the two measures or is it pointing to the different processing of velocity and the trajectory interference magnitude. Please discuss about this point.

Again, thank you for this interesting observation. This point is now mentioned to the discussion of this issue with a careful call for a replication (p. 35).

To elaborate on the issue, response time and maximum deviation correlate fairly well in our data (r ranging between 0.5 and 0.7 depending on the stimulus type), but not to the point of assuming that velocity is approximately constant. Those correlations reflect that there is a fair share of noise across mouse movements (within stimulus types).

As pointed out by R2, across stimulus type, there seem to be differences in velocity between colour-associated incongruent and non-response set items. Both condition have a similar mean maximal deviation but response times in the former are shorter (approx. at the level of color-neutral items), indicating faster velocity. This leads to this inverse pattern with semantic conflict and semantic relevance. Within those stimulus types, correlations between response times and maximal deviation are very similar. Depending on whether we calculate correlations on all data, or calculate the correlation for each participant and average them:

colour-associated incongruent: r = 0.59 and r = 0.64

non-response set: r = 0.57 and r = 0.63

This may indicate a similar noise level in those conditions (if we interpret it correctly), although this analysis is quite crude. This might point towards differences in response selection and execution when processing those stimuli, although this result should be replicated.

Finally, we did not report velocity measures in the MS because of the high number of measures presented, but also because in conditions where strict temporal deadlines are imposed to the participants, velocity becomes difficult to interpret. For instance, faster maximum velocity can be observed in easy trials (e.g., congruent trials), but also in difficult trials where participants tend to speed their movements for responding in time.

If in light of those details R2 sees an issue that we should discuss more thoroughly, we would be happy to do it.

3) The part describing the analysis was very difficult to understand. For example, for line 327, when the authors say, “above and below direct path”, what is up and what is down? I think it can be explained more clearly, perhaps including one schematic figure for it.

The version of Figure 1 (now Figure 2) included in the original submission missed some information (mentioned in the caption) on the deviation and x-coordinates measures, due to an error with the generation of the rasterised file. The correct Figure is now included.

However, thanks to your comment, we further adapted this Figure make the description of the deviation measures clearer. We clearly separated the max deviation from the deviation/x-coordinates calculated in the normalized time analyses. We also provide an example of a time sample (the 3rd latest in the trajectory) which is below the direct path (therefore with a negative deviation). We directly refer to this time sample for illustrating the sentence Reviewer 2 pointed out as unclear (p.15).

Additionally, we slightly modified or reformulated several sentences describing the mouse-tracking measures for improving clarity in the same section.

6. PLOS authors have the option to publish the peer review history of their article ([what does this mean?](https://journals.plos.org/plosone/s/editorial-and-peer-review-process#_blank)). If published, this will include your full peer review and any attached files.


If you choose “no”, your identity will remain anonymous but your review may still be made public.


**Do you want your identity to be public for this peer review?** For information about this choice, including consent withdrawal, please see our [Privacy Policy](https://www.plos.org/privacy-policy).

Reviewer #1: No

Reviewer #2: No

[NOTE: If reviewer comments were submitted as an attachment file, they will be attached to this email and accessible via the submission site. Please log into your account, locate the manuscript record, and check for the action link "View Attachments". If this link does not appear, there are no attachment files.]

While revising your submission, please upload your figure files to the Preflight Analysis and Conversion Engine (PACE) digital diagnostic tool, <https://pacev2.apexcovantage.com/>. PACE helps ensure that figures meet PLOS requirements. To use PACE, you must first register as a user. Registration is free. Then, login and navigate to the UPLOAD tab, where you will find detailed instructions on how to use the tool. If you encounter any issues or have any questions when using PACE, please email PLOS at [figures@plos.org](mailto:figures@plos.org). Please note that Supporting Information files do not need this step.
